# Supplementary material for: Activity of Human-Specific Interlaminar Astrocytes in a Chimeric Mouse Model of Fragile X Syndrome
Source: Int J Mol Sci. 2025 Jul 6;26(13):6510. doi: 10.3390/ijms26136510 (PMC12250119; doi:10.3390/ijms26136510)
Supplement: Supplementary file 1 [file ijms-26-06510-s001.zip › ijms-3667846-supplementary.pdf]

Supplemental Figure S1: Enhanced ATP-evoked Ca^{2+} signaling in FXS astrocytes at 4 months

(A) Time-lapse imaging of CTR and FXS astrocytes in a cortical slice from a 4-month-old chimeric mouse. Images of RFP-expressing astrocytes (red) and time-lapse images of the fluo-4 channel (green) showing ATP-evoked Ca^{2+} responses (top panel: CTR, bottom panel: FXS). Traces for the Ca^{2+} responses are shown for the regions of interest (ROI 1 and 2) indicated in the RFP images. **(B)** Peak amplitude of ATP-evoked Ca^{2+} responses is higher in FXS astrocytes. Mann-Whitney test. **$P < 0.01$. N = 6-8 slices from 4-5 mice in each group.

Supplemental data movie S1: A movie of ATP-evoked calcium signaling in ILAs in cortical slices from a 6 month old chimeric mouse engrafted with hi-Astrocytes expressing GCaMP6f. Calcium events detected by AQUA in a single process are overlayed.

Supplemental data movie S2: A movie from a 6 month old chimeric mouse engrafted with hi-Astrocytes expressing GCaMP6f, imaged through a cranial window while awake and head restrained.
