# Supplementary figures and images for: Activity of Human-Specific Interlaminar Astrocytes in a Chimeric Mouse Model of Fragile X Syndrome
Source: Int J Mol Sci. 2025 Jul 6;26(13):6510. doi: 10.3390/ijms26136510 (PMC12250119; doi:10.3390/ijms26136510)

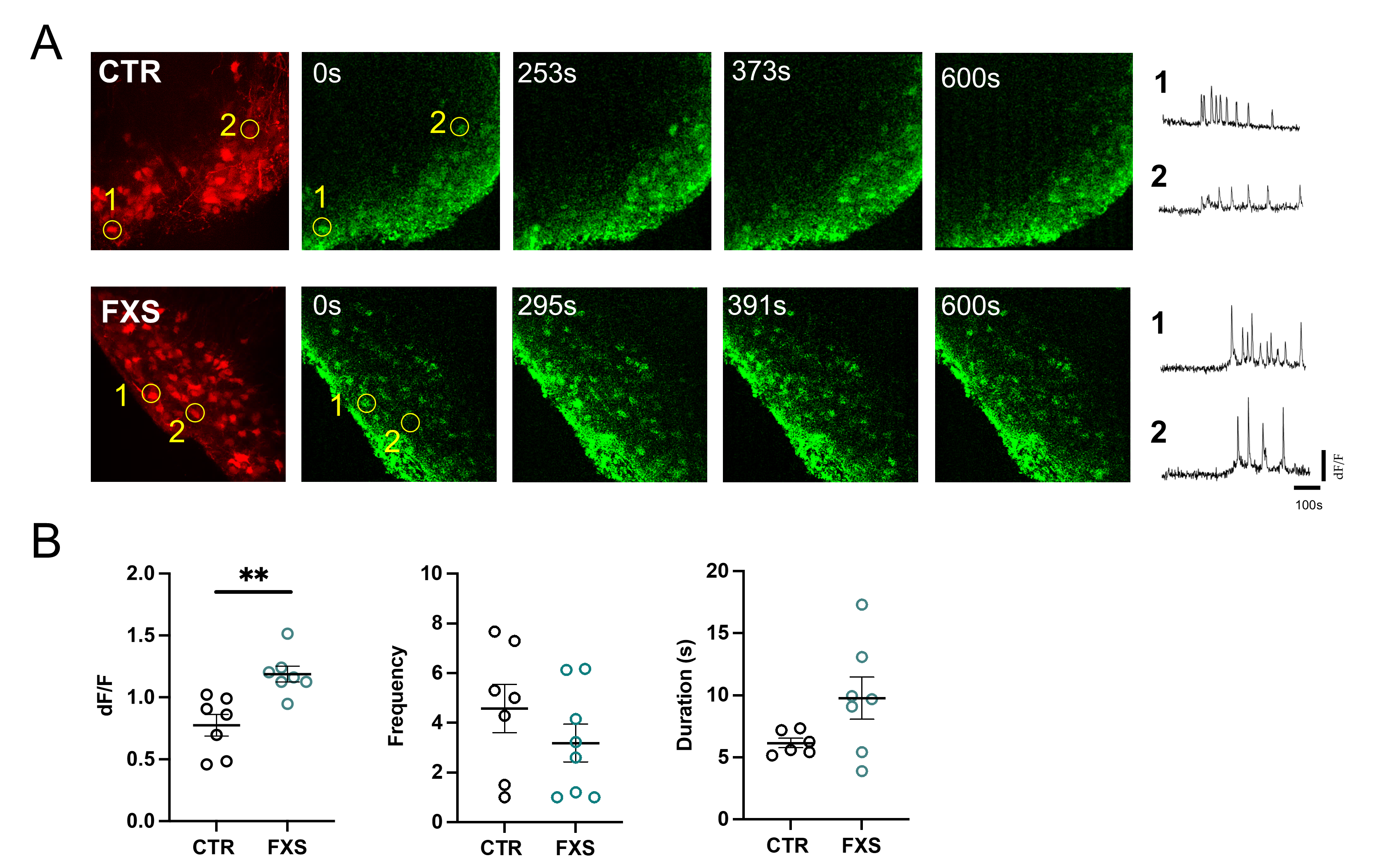

Supplement: Supplementary file 1 [file ijms-26-06510-s001.zip › ijms-3667846-Supp Figure S1 Ca imaging slices ILA soma.tif]
